# Glial fibrillary acidic protein astrocytopathy and tuberculous meningoencephalitis occurring in a patient with *Legionella* pneumonia: a case report

**DOI:** 10.1186/s12883-023-03113-w

**Published:** 2023-02-13

**Authors:** Ke Li, Jingwei Wu, Junwu Chen, Yong You

**Affiliations:** 1 Department of Neurology, The Second Affiliated Hospital of Hainan Medical University, 570013 Haikou, China; 2 Department of Internal Medicine, Changjiang County Medical Group, Changjiang, China

**Keywords:** Glial fibrillary acidic protein, Tuberculous, Meningoencephalitis, *Legionella*

## Abstract

**Background:**

Autoimmune glial fibrillary acidic protein (GFAP) astrocytopathy is a recently identified recurrent meningoencephalomyelitis with GFAP immunoglobulin G presence in the serum or cerebrospinal fluid (CSF) as a specific biomarker. GFAP astrocytopathy is closely associated with the occurrence of some tumors and often coexists with other antibodies, such as the N-methyl-D-aspartate receptor and aquaporin-4 antibodies. However, GFAP astrocytopathy complicated by central nervous system infection is rare.

**Case presentation:**

Here, we present the case of a patient admitted to a local hospital due to a prominent fever and cough. The patient had a 1-month history of headaches before admission that were not considered serious at the time. Metagenomic next-generation sequencing (mNGS) of bronchoalveolar lavage fluid revealed a high sequence number of *Legionella pneumophila* and a few mycobacteria. His cough and fever improved significantly after antibiotic treatment. Still, a slight headache remained. Subsequently, his condition worsened, and he visited our hospital with a disturbance of consciousness. *Mycobacterium tuberculosis* was detected with mNGS of the CSF, while the CSF and serum were also positive for GFAP antibodies. Following anti-tuberculosis and steroid therapy, the patient’s symptoms improved, and he tested negative for the GFAP antibody.

**Conclusion:**

This is the first reported case of GFAP astrocytopathy complicated by tuberculous meningoencephalitis. Due to overlaps in the clinical manifestations of the two diseases, GFAP astrocytopathy is sometimes misdiagnosed as tuberculous meningoencephalitis. Therefore, in addition to ensuring careful identification of the two diseases, clinicians need to be aware of their possible co-existence.

## Background

Autoimmune glial fibrillary acidic protein (GFAP) astrocytopathy, first defined in 2016, is an autoimmune disease of the nervous system, with presence of GFAP immunoglobulin G (IgG) in the serum or cerebrospinal fluid (CSF) as a specific biomarker [1]. Most patients have an acute or subacute onset of the disease. The main manifestations are involvement of the meninges, brain, spinal cord, and optic nerve [1, 2]. Cerebral linear perivascular radial gadolinium enhancement in the white matter perpendicular to the ventricle is the most characteristic feature observed during magnetic resonance imaging (MRI) [3]. Lesions can also occur in the subcortical white matter, basal ganglia, hypothalamus, brainstem, cerebellum, and spinal cord [2]. Most patients with GFAP astrocytopathy respond well to steroid therapy, although relapses and death can also occur.

Previous studies have found that GFAP astrocytopathy is closely associated with the occurrence of some tumors and often coexists with other antibodies, such as the N-methyl-D-aspartate receptor (NMDAR) and aquaporin-4 (AQP4) antibodies [2]. However, GFAP astrocytopathy complicated by central nervous system infection is rare. Here, we report a novel case of a patient with GFAP astrocytopathy complicated by tuberculous meningoencephalitis. Following anti-tuberculosis and steroid therapy, the patient’s symptoms improved, and he tested negative for the GFAP antibody.

## Case presentation

**Fig. 1:**
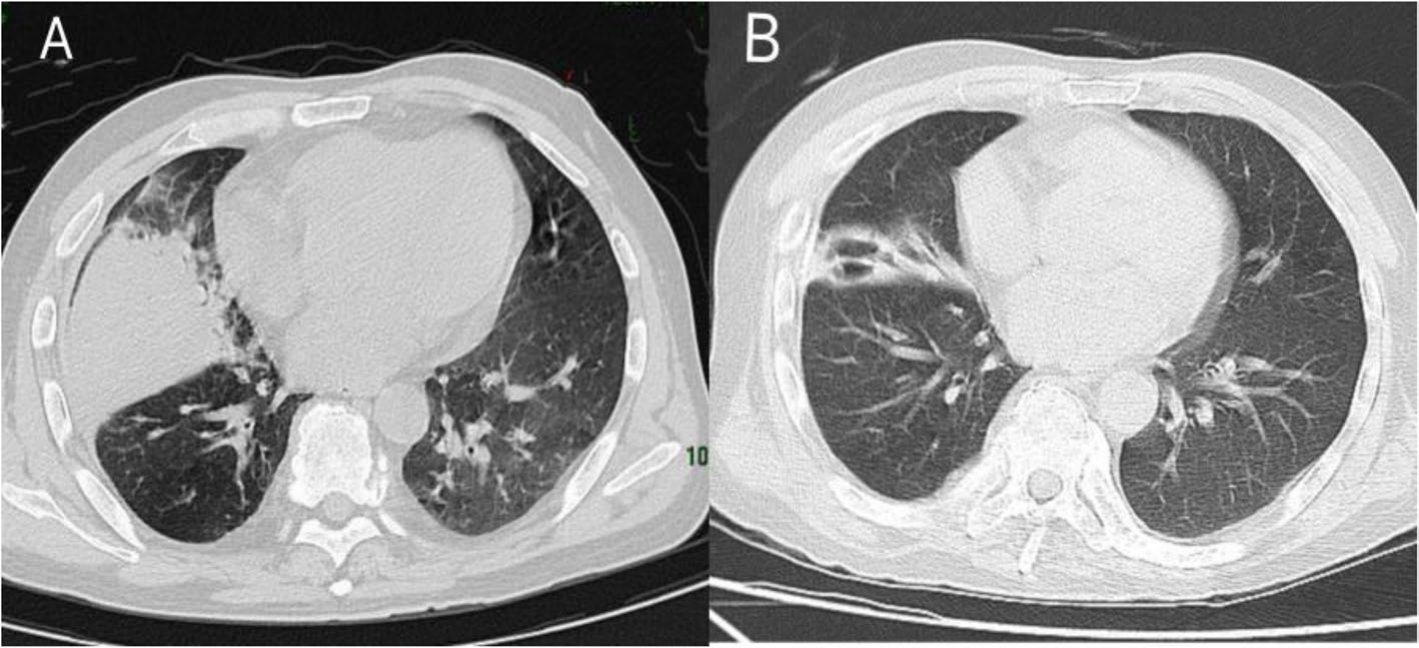
Chest computed tomography (CT) scan in the early stage showed right lower lobe consolidation, multiple calcifications in the bilateral perihilar, and small mediastinal lymph nodes **A**. Inflammation of the right lung was mostly cleared after antibiotic treatment (**B**)

A 45-year-old man with symptoms of cough and fever was admitted to the respiratory medicine department of a local hospital. The patient reported a 1-month history of headaches before admission but was otherwise healthy. Temperature was not measured before admission. On admission, his temperature was 39.0 °C, with oxygen saturation kept at 95% while breathing ambient air. His respiratory rate was 24 breaths/min, and his level of consciousness remained undisturbed. Chest auscultation revealed coarse crackles in both lower lungs. Chest computed tomography (CT) scan showed right lower lobe consolidation, multiple calcifications in the bilateral perihilar, and small mediastinal lymph nodes (Fig. 1A). Metagenomic next-generation sequencing (mNGS) of bronchoalveolar lavage fluid indicated *Legionella pneumophila* (8702 unique reads) as the pathogenic microorganism while the presence of a few mycobacteria (9 unique reads) was also observed. He was subsequently diagnosed with *Legionella* pneumonia and was treated with piperacillin-tazobactam sodium and levofloxacin. After two weeks, the patient’s temperature gradually returned to normal, and his headache slightly improved.

**Fig. 2:**
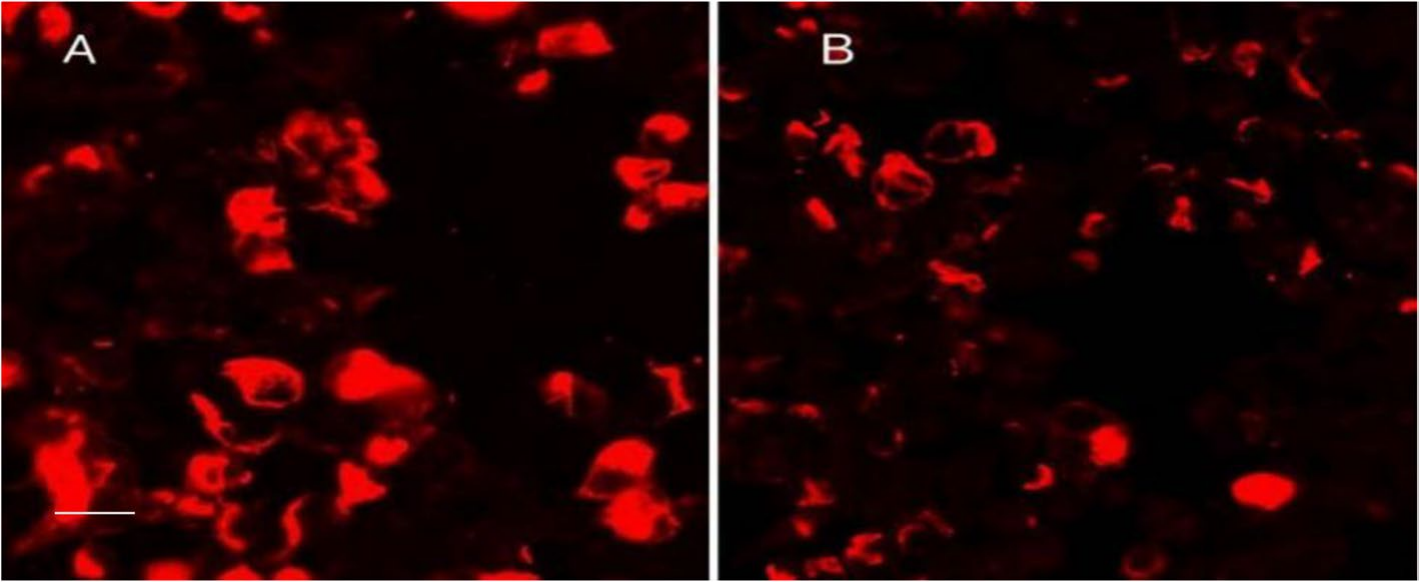
Anti-GFAP in cerebrospinal fluid (CSF) and serum validated by a transfected-cell-based indirect immunofluorescence test. Tests for CSF GFAP-IgG (1:100) and GFAP-IgG (1:32) were positive, as shown in A and B, respectively. (scale bar = 50 μm)

**Fig. 3:**
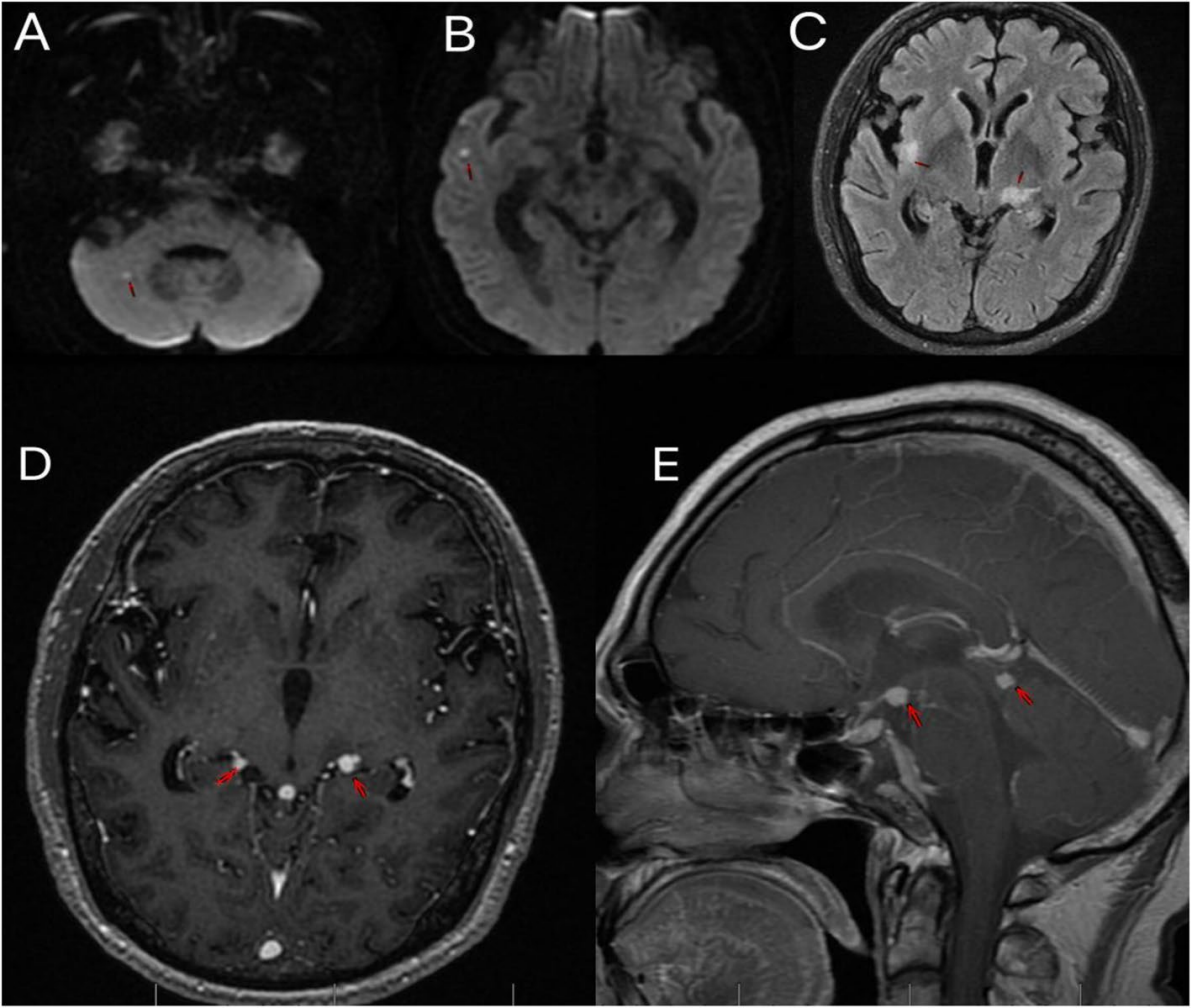
**A**-**B**: Axial Diffusion-Weighted Imaging (DWI) revealing punctate hyperintense lesions in the right cerebellar hemispheres and the temporal lobe. **C**: Axial T2-Weighted-Fluid-Attenuated-Inversion-Recovery (FLAIR) imaging revealing hyperintense lesions in the right insula and the left thalamus. **D**: Axial T1-Weighted contrast-enhancement imaging revealing significant circle enhancement lesions in the ambient cistern and quadrigeminal cistern. **E**: Sagittal T1-Weighted contrast-enhancement imaging revealing significant enhancement lesions in the ambient cistern and prepontine cistern

One week after discharge, the patient developed a decreased state of consciousness, and his temperature was 37.8 °C. On physical examination, the patient had a decreased level of consciousness and neck resistance, with bilateral Kernig’s sign positivity. The bilateral eye movements were slightly limited. Bilateral muscle strength testing of the upper and lower limbs indicated patient muscle strength at grade 4. Chest CT showed that the inflammation of the right lung was mostly cleared when compared with the previous CT scan (Fig. 1B). CSF pressure significantly increased to 330 mmH2O. CSF analysis revealed a slightly increased number of white blood cells (65*10^6/L), increased protein (1381 mg/L), and a reduction in glucose (2.16 mmol/L) and chloride (110.8 mmol/L) levels. CSF ADA levels (23 U/L) were also increased. mNGS of the CSF revealed *Mycobacterium tuberculosis* as the pathogenic microorganism (52 unique reads). Tests for GFAP-IgG in the CSF (1:100) and serum (1:32, analyzed by a fixed cell-based assay, examined by confocal fluorescence microscopy; Fig. 2) were positive. Serological examination results were negative for onconeural antibodies, including ANNA-1, NMDAR, AMPH, Yo, CV2, PNMA2, Ri (ANNA‐2), recoverin, GAD65, and Tr (DNER) antibodies. Tests for serum and CSF AQP4 and MOG antibody were also negative. Brain MRI revealed punctate infarcts in the right cerebellar hemispheres and temporal lobe and multiple lesions in the right insula, left thalamus and around the cistern. Some of the nodules were enhanced after injection of gadolinium (Fig. 3). Spinal cord MRI (including the cervical, thoracic, and lumbar spine) was normal. Therefore, the patient was diagnosed with GFAP astrocytopathy and tuberculous meningoencephalitis. He was then administered isoniazid 0.6 g/day, rifampicin 0.6 g/day, pyrazinamide 1.5 g/day, ethambutol 0.75 g/day, and levofloxacin 0.5 g/day for anti-tuberculosis treatment. Simultaneously, he was treated with 250 mg/day of intravenous methylprednisolone for 5 days, followed by 120 mg/day of intravenous methylprednisolone for another three days, which was eventually changed to oral prednisone tablets (60 mg) as maintenance therapy. The patient’s consciousness gradually improved, and the headaches improved significantly. After one month, the patient’s CSF pressure dropped to 170 mmHg. Re-examination of the CSF showed reduced number of white blood cells (24*10^6/L) and protein (530 mg/L). CSF glucose and chloride levels returned to normal. GFAP antibody levels in the serum and CSF were negative at the 12-month follow-up visit. There was no relapse after the treatment.

## Discussion and conclusion

The patient was admitted to a local hospital due to a prominent fever and cough. Although a few mycobacteria were found in the bronchoalveolar lavage fluid through mNGS, combined with the patient’s symptoms, lung CT, and mNGS results of bronchoalveolar lavage fluid, he was diagnosed early with *Legionella* pneumonia, which is the main clinical manifestation of Legionnaires’ disease. This disease is a systemic infectious disease caused by gram-negative bacteria. In addition to typical pneumonia, nervous system involvement is a common extrapulmonary manifestation. It is usually manifested with the presence of headaches. In severe cases, disturbance of consciousness, epileptic seizures, and cranial nerve palsy can also be observed. Therefore, despite the presence of early-stage headaches, it can easily be mistaken for a non-specific manifestation of Legionnaires’ disease. This can lead respiratory physicians in local hospitals to neglect the possible presence of a neurological disease, resulting in misdiagnoses and delaying appropriate early diagnosis and treatment. In this case, the patient’s headache was relieved after antibiotic treatment at a local hospital; however, mild headache after discharge remained. Subsequently, the patient’s condition worsened, and he developed disturbance of consciousness. CSF examination results were consistent with the characteristics of tuberculous meningoencephalitis. Finally, diagnosis of tuberculous meningoencephalitis was confirmed with mNGS of the CSF, which revealed the presence of *Mycobacterium tuberculosis*.

The difference between this report and other cases of simple central nervous system infection is that the patient was also positive for GFAP-IgG that turned negative after steroid therapy. Novel GFAP-IgG autoantibodies produce an astrocyte-restricted staining pattern and are identified as an autoimmune GFAP astrocytopathic biomarker. Autoimmune GFAP astrocytopathy is a recurrent meningoencephalomyelitis that has been identified in recent years. Its symptoms are diverse and non-specific and are related to the location and extent of the lesion. The typical clinical features are subacute onset of encephalitis, meningitis, myelitis, or a combination of these syndromes. The first symptoms are usually headaches accompanied with fever, disturbance of consciousness, seizures, and mental symptoms. It can also present as an isolated area postrema syndrome [4]. Typically, the subacute headache is prominent. A 2016 study by Flanagan et al. found that 17 out of 38 patients with GFAP astrocytopathy had a typical linear perivascular radial gadolinium pattern enhancement, while 7 had normal imaging results [3]. Recently, some case reports have shown that, in addition to common site lesions, such as the subcortical white matter, basal ganglia, hypothalamus, brainstem, cerebellum, and spinal cord, GFAP astrocytopathy can also manifest as hypertrophic meningoencephalitis [5] or mild encephalitis with reversible splenial corpus callosum lesions [6]. This patient had no typical linear lesions, but the CSF GFAP antibodies were highly specific and sensitive and led to the diagnosis of GFAP astrocytopathy. GFAP-IgG can be produced by peripheral and CNS infiltrating lymphoid cells. A previous study by Fang et al. suggested that GFAP-specific IgG status is ascertained with more sensitivity in the CSF than in the serum. Moreover, CSF analysis yielded no false-positive results on GFAP-transfected cells [1].

To date, this is the first reported case of GFAP astrocytopathy complicated with tuberculous meningoencephalitis. Nearly 40% of patients with GFAP astrocytopathy have prodromal symptoms, such as runny nose and sore throat; however, coexistence of GFAP astrocytopathy and an intracranial infection is rare. Furthermore, previous studies have ever found evidence of herpes simplex virus infection in patients with GFAP astrocytopathy [7–9]. Of the 19 patients included in the study by Long et al., 2 showed evidence of herpes simplex infection at the time of onset [7]. The cases reported by Li et al. [8] and Handoko et al [9]. were both diagnosed with GFAP astrocytopathy after herpes simplex infection. Still, the relationship between viral infection and GFAP astrocytopathy is unclear. He et al. also reported a case of GFAP astrocytopathy following *Brucella* infection [10], where they suggested that some pro-inflammatory mediators are released after pathogenic infection, leading to disruption of astrocyte function and the blood-brain barrier. GFAP in astrocytes has the potential to expose self-epitopes and possibly alter its subcellular distribution through proteolytic cleavage, phosphorylation, or dephosphorylation. Subsequently, a large number of immune cells from the peripheral circulation enter the central nervous system, causing a series of immune responses [11–13]. Other infectious diseases have not yet been found to cooccur with GFAP astrocytopathy. In this case, the patient was infected with *Mycobacterium tuberculosis* and *Legionella pneumophila*, both of which are capable of invading the body when the patients are immunocompromised. At present, the order of occurrence of the three diseases in this patient cannot be determined. Therefore, further research is needed to confirm whether the patient’s immune disorder caused infection susceptibility or whether the infections induced the immune dysfunction. This case provides new clinical data on GFAP astrocytopathy, which can help to further explore the underlying pathophysiological mechanisms between infection and GFAP astrocytopathy.

In conclusion, this is the first reported case of GFAP astrocytopathy complicated by tuberculous meningoencephalitis. There are some overlaps in the clinical manifestations of the two diseases, leading to GFAP astrocytopathy sometimes being misdiagnosed as tuberculous meningoencephalitis. Therefore, in addition to ensuring carefully identification of the two diseases, clinicians need to be aware of their possible co-existence.

## Data Availability

The datasets presented in this article are not readily available due to ethical and privacy restrictions. Requests to access the datasets should be directed to the corresponding author.

## Abbreviations

ADA: adenosine deaminase
AQP4: aquaporin-4
CSF: cerebrospinal fluid
CT: Chest computed tomography
DWI: Diffusion-Weighted Imaging
FLAIR: Fluid-Attenuated-Inversion-Recovery
GFAP: Autoimmune glial fibrillary acidic protein
IgG: immunoglobulin G
mNGS: Metagenomic next-generation sequencing
MRI: magnetic resonance imaging
NMADR: N-methyl-D-aspartate receptor
